# Assessement of a structured technological support intervention on uptake of video consultations

**DOI:** 10.1192/bjo.2021.540

**Published:** 2021-06-18

**Authors:** Tejas Kotwal, Kerushan Thomas, Carlos Escudero King, Avirup Gupta

**Affiliations:** 1 king's college london; 2 South London and Maudsley NHS Foundation Trust

## Abstract

**Aims:**

The coronavirus pandemic has led to an increased reliance on remote patient-clinician interactions, mainly the use of telephone and video consultations. Video consultations are key in psychiatric care, as telephone appointments do not sufficiently allow clinicians to accurately ascertain a patient's mental status and perform a risk assessment. The aim of our quality improvement project was to increase the uptake of video consultations within a community mental health team, focusing on substituting telephone consultations for video.

**Method:**

We accessed Electronic Patient Records to retrospectively quantify the method of contact for 130 consultations delivered over a 4-week period. After collecting baseline data, we conducted focused interviews with 10 care providers, identifying the specific clinician and patient barriers to video uptake that informed our intervention design.

Our intervention consisted of two 4-week Plan, Do, Study, Act (PDSA) cycles.

PDSA 1 involved delivering a focused PowerPoint presentation to the care team, highlighting the benefits of video consultation technology and encouraging clinicians to use it as their primary method of remote communication with patients. Additionally, we conducted qualitative interviews with members of the team to highlight the successes and challenges thus far.

PDSA 2 involved creating a video consultation instructional PDF which highlighted how to operate the technological aspects of both Microsoft Teams and WhatsApp Video Call. This included: how to set-up video calls, accept invitations, and overcome common troubleshooting issues.

The proportion of remote consultations was quantified retrospectively to compare trends in video consultation uptake from baseline to the conclusion of PDSA 2.

**Result:**

Overall, we saw a 15% increase in video consultations with respect to baseline. The greatest change was attributable to PDSA cycle 1, which incurred an 8% increase in video consultation uptake, from 13.85% to 21.9%. PDSA cycle 2 further increased video consultation uptake by 6.97%, from 21.9% to 28.87%. Specifically focusing on remote consultations, the proportion conducted with video rather than telephone increased by 17.3%. Interviewed clinicians reported limited financial access, technological fluency, and issues with patient privacy as the most important barriers to the uptake of video consultations.

**Conclusion:**

Our project successfully increased the proportion of consultations conducted by video. This was achieved by targeting interventions to address both patient and clinician barriers to video consultation uptake. Moreover, we understand that motivating and mobilising the care team was a key factor. Possible future work includes improving the sustainability of the interventions and assessing their efficacy in other care teams.

